# Holographic Grating Enhancement of TI/PMMA Polymers in the Dark Diffusion Process

**DOI:** 10.3390/polym13111735

**Published:** 2021-05-26

**Authors:** Peng Liu, Xiudong Sun

**Affiliations:** 1 College of Physics and Electronic Engineering, Sichuan Normal University, Chengdu 610101, China; 2 Institute of Modern Optics, School of Physics, Key Laboratory of Micro-Nano Optoelectronic Information System, Ministry of Industry and Information Technology, Key Laboratory of Micro-Optics and Photonic Technology of Heilongjiang Province, Harbin Institute of Technology, Harbin 150001, China; xdsun@hit.edu.cn; 3 Collaborative Innovation Center of Extreme Optics, Shanxi University, Taiyuan 030006, China

**Keywords:** dark diffusion, photopolymerization, holographic grating enhancement, pre-exposure, post-exposure, TI/PMMA photopolymer

## Abstract

The dark diffusion enhancement process (DDEP) caused by photopolymerization during the pre-exposure of TI/PMMA (titanocene dispersed methyl methacrylate matrix) polymers was theoretically analyzed and experimentally investigated, revealing the holographic grating enhancement of TI/PMMA polymers in the post-exposure process without additional operations. The diffusion of photo-initiators and photoproducts dominated the grating enhancement process after exposure. We adopted two pre-exposure methods, long-time (second level) and short-time (millisecond level) laser exposure, at 532 nm, to investigate the DDEP during the post-exposure process. A five-fold enhancement in grating strength was achieved in consecutive long-time pre-exposures, while a two-fold grating development was examined after short-time exposure. Additionally, the exposure durations and repetition rates influenced the grating increment of the DDEP. This study provided a basis for the feasibility of holographic application in TI/PMMA photopolymers via the dark diffusion effect.

## 1. Introduction

Volume holographic recording is a type of three-dimensional storage technology [1,2,3]. Compared to traditional two-dimensional storage, such as magnetic storage [4], optical storage [5] and semiconductor storage [6], holographic storage exhibits the excellent properties of a high transmission rate, high storage density and simultaneous reading/writing ability [7,8]. The performance of volume holographic storage is mainly determined by the recording medium. In many recording materials, photopolymers exhibit comprehensive holographic properties alongside a low price and simple fabrication [9,10,11]. TI/PMMA (titanocene dispersed methyl methacrylate matrix) polymers have been developed based on the traditional PQ/PMMA (phenanthrenequinone dispersed methyl methacrylate matrix) polymer fabrication method, named thermo-polymerization [12]. The difference between the two polymer components is the photo-initiator. We replaced PQ (phenanthrenequinone) molecules [13] with TI molecules [14,15] to create a new-generation photo-initiator. This kind of photopolymer exhibits better holographic properties than that of PQ/PMMA [16]. Meanwhile, TI/PMMA polymers can record holographic intensity and polarization modulated gratings through long-time (second level) and short-time (millisecond level) exposure [17,18,19], exemplifying the competitiveness of holographic storage applications.

Grating strength enhancement caused by dark diffusion is a unique characteristic of photopolymers during the holographic recording process. Many investigations have proved this behavior in photopolymers, such as PQ/PMMA [20,21] and PVA/AA (poly (vinyl alcohol)-acrylamide) [22] materials. However, dark diffusion in the TI/PMMA polymer has not yet been investigated. The essence of the dark diffusion enhancement process (DDEP) is the diffusion of photo-initiators and photoproducts after exposure [21]. By studying this special characteristic, the holographic recording period can be reduced and the exposure energy availability improved. In this article, the DDEP performance of TI/PMMA polymers during the post-exposure process was investigated using two recording approaches. On one hand, we adopted long-time exposure (second level) to record gratings. In the experiment, the influence of exposure density and pre-exposure time on DDEP was firstly examined. Then, we studied the recording ability of a single grating and multiplexed gratings on the DDEP. On the other hand, a short exposure (millisecond level) was used in the pre-exposure period. The effects of a short duration and various repetition rates were analyzed experimentally. All the results indicated that TI/PMMA polymers could generate dark diffusion and exhibit excellent performance.

## 2. Materials and Methods

### 2.1. Materials and Preparations

**Figure 1:**
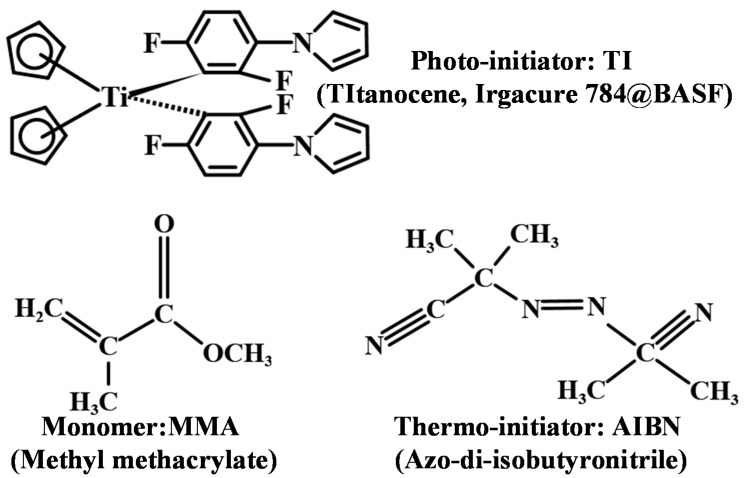
Chemical structures of the main solutes and solvent in TI/PMMA photopolymer samples.

Here, we fabricated TI/PMMA polymers using a three-step polymerization method [14], which included pre-polymerization, high-temperature treatment and low-temperature polymerization. The chemical structures of the main solutes and solvents used in the fabrication of TI/PMMA polymers are depicted in Figure 1 [23,24,25]. The solvent MMA was used as the monomer in the TI/PMMA matrix, which contributed to the polymerization process through photo-initiation and thermo-initiation. The TI and AIBN (azo-di-isobutyronitrile) molecules acted as photo-initiators and thermo-initiators, respectively, to activate the polymerization reaction. AIBN generated free radicals under heating conditions, which initiated the chain polymerization of MMA monomers. TI molecules were excited by laser exposure to produce free radicals that polymerized the surrounding monomers. This process modulated the refractive index of the exposure area to generate holographic grating. Optimized doping concentrations of a photo-initiator (TI molecules 4.0 wt. %) and thermo-initiator (AIBN molecules 2.0 wt. %) were introduced into the substrate MMA monomers, which was investigated on our previous page [14]. The mixture was stirred at 40 °C for 24 h to make the solute dissolve completely and remove the nitrogen generated by the thermo-decomposition of the AIBN. Then, the solution was placed in the incubator (TAISITE, Tianjing, China) at 72 °C for 15 min to intensify the thermal polymerization reaction. After this high-temperature treatment the mixture became viscous, and after a slow process of low-temperature polymerization at 45 °C for 48 h, the viscous mixture changed into a lumpy solid state. Samples with different thicknesses, of between 1 and 3 mm, were prepared using different molds and subsequent polishing operations. We used TI/PMMA materials of the same concentration ratio to measure the dark diffusion enhancements of single and multiplex grating recordings after pre-exposure.

### 2.2. Holographic Setup

**Figure 2:**
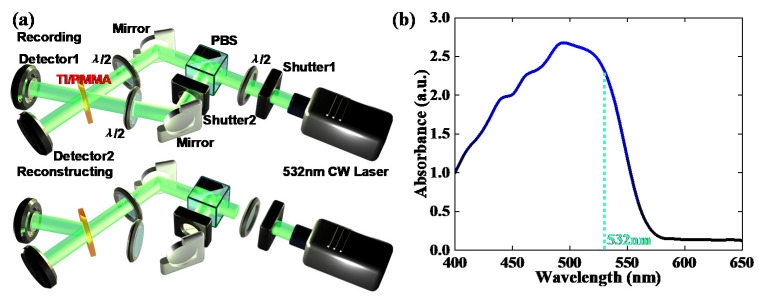
(**a**) Recording and reconstructing approaches for holographic measurement, (**b**) light absorption spectrum of TI/PMMA polymers at the range of 400–650 nm.

During the holographic recording step, an interference system comprising of a two-wave coupling optical path was used to measure holographic parameters [26,27], as shown in Figure 2a. At the recording stage, we opened Shutter2 to ensure that the two beams were coupled. The half-wave plate before the PBS controlled the intensity ratio of the two beams, while the half-wave plates after the PBS adjusted the polarization direction of the two beams. In our experiment, two recording beams were tuned to parallel polarizations. Shutter1 was applied to control the exposure flux transmitting to the sample. Then, during the reconstructing process, we closed Shutter2 to prevent two-wave coupling. The reconstructing beam transected the sample along the same path as the recording beam in order to reconstruct the diffracted beam. Shutter1 remained in an off state during the DDEP, only periodically opening for 0.5 s to read the diffracted intensity with lower exposure energy to avoid the influence of light absorption on diffraction efficiency. According to Figure 2, we can see that the light absorption peak of the TI/PMMA polymer was around 500 nm at the range of 400–650 nm. In order to avoid an excessive scattering effect and to enable consideration of the samples with the existing laser in the laboratory, we chose a 532 nm laser as the excitation source. Hence, a solid state 532 nm green laser with a maximum exposure energy of 115 mW/cm^2^ was adopted to investigate the dark diffusion properties of the TI/PMMA polymers. The intersection angle between the two recording beams was 10 degrees. By placing an electronical rotator at the bottom of the TI/PMMA sample holder, the multi gratings recorded by angle multiplexing in the same exposure area could be realized. Diffraction efficiency and grating strength were the common parameters used to describe the index of the holographic grating. Diffraction efficiency was defined as the ratio of the diffracted intensity to the transmitted intensity of the reconstructing beam, and grating strength was defined as the square root of the diffraction efficiency.

## 3. Results and Discussion

### 3.1. Theoretical Analysis on Dark Diffusion Enhancement Process (DDEP)

There are two main processes in holographic grating formation: the photochemical and photophysical reactions. The photoinitiated molecule absorbs photons to generate radicals, which can further initiate polymerization reactions with the surrounding monomers. This is the main photochemical process. Meanwhile, the photophysical reaction is the diffusion effect of photoinitiated molecules due to the concentration difference between dark fringe and bright fringe regions caused by interference exposure [28]. In the system of TI/PMMA polymers, TI represents the photoinitiated molecules, while PMMA stands for monomers. Here, we introduced the absorption coefficient influenced by sample thickness and exposure time into the traditional non-local diffusion model [17,21]. The temporal and spatial relations of photopolymerization and dark diffusion have been determined, as shown below [29,30]:

(1) $$\left[\mathrm{Photop}\mathrm{roduct}](x,t)=\frac{1}{\varepsilon d}\ln\{1+[\exp(\varepsilon \mathrm{TI}d)-1]\exp[-\varepsilon d\phi I_{0}(1+V\cos Kx)t]\right\}$$

(2) $$D_{\mathrm{TI}}(x,\tau )=\frac{{\mathrm{TI}}_{0}}{2}(1+V\cos Kx)\exp(-fk_{d}\tau )\exp(-\alpha d)$$

(3) $$D_{\mathrm{Photoproduct}}(x,\tau )=P\mathrm{hotop}r{\mathrm{oduct}}_{0}-D_{\mathrm{TI}}(x,\tau ){=\mathrm{TI}}_{0}-D_{\mathrm{TI}}(x,\tau )$$

where *d* stands for the thickness of sample and ϕ is the quantum yield, while ε represents the molar absorption coefficient, I_0_ is the exposure intensity, V is the light visibility and $fk_{d}$ depicts polymerization rate. TI_0_, Photoproduct_0_ and TI describe the original concentration of the TI molecules before exposure, the concentration of the photoproduct, and the TI molecules after exposure, respectively. Equation (1) expresses the photopolymerization in pre-exposure, a photoinitiated polymerization reaction in which TI photo-initiators and PMMA monomers polymerized to form photoproducts. While the dark diffusion is depicted in Equations (2) and (3), $D_{\mathrm{TI}}(x,\tau )$ and $D_{\mathrm{Photoproduct}}(x,\tau )$ describe the diffusion coefficient of the TI molecules and the photoproducts caused by the concentration difference between the bright and dark fringe regions in the exposure area.

**Figure 3:**
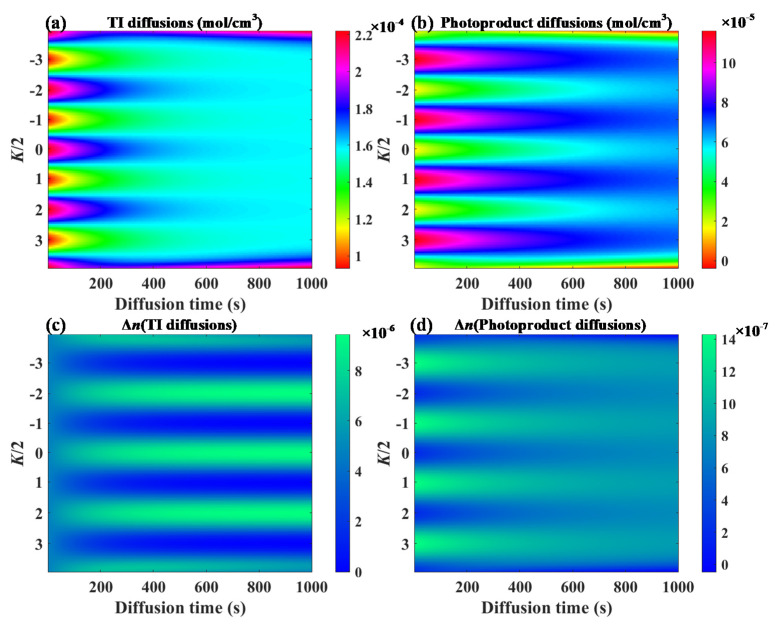
Theoretical model of DDEP: (**a**) TI diffusion distributions, (**b**) photoproduct diffusion distributions, (**c**) refractive index modulation of TI diffusion, (**d**) refractive index modulation of photoproduct diffusion.

The whole grating formation of the DDEP included three stages: photoinitiated polymerization, dark diffusion of the photo-initiator, and the reverse diffusion of photoproducts. During the second step, the diffusion of the photo-initiator, TI molecules (from the dark fringe to bright fringe regions), were the main requirement for improving the grating strength, while the third step, the reverse diffusion of photoproducts (from the bright fringe to dark fringe regions, named anti-diffusion), could attenuate the holographic grating. Figure 3 depicts the diffusion distributions and refractive index modulations of the TI molecules and photoproducts during the dark diffusion process. The refractive index modulation was calculated using Equation (4) [31], where N_TI_ and N_Photoproduct_ are the constants related to the refractive indices of TI molecules and photoproducts. Here, the refractive indexes of the TI molecules, photoproducts and substrates were 1.6298, 1.5005 and 1.4760, respectively. It was possible to improve the refractive index of the TI concentration gradient by the order of 10^−6^, while the distribution of the photoproduct could only be influenced by a level of 10^−7^. Overall, the DDEP improved holographic grating strength following a long diffusion due to the refractive index modulation of the TI molecules.

(4) $$\Delta n(\tau )=N_{\mathrm{TI}}[\mathrm{TI}](\tau )-N_{\mathrm{Photoproduct}}[\mathrm{Photoproduct}](\tau )$$

### 3.2. The Dark Diffusion Enhancement Process (DDEP) in TI/PMMA Polymers with Consecutive (Long-Time) Exposure

**Figure 4:**
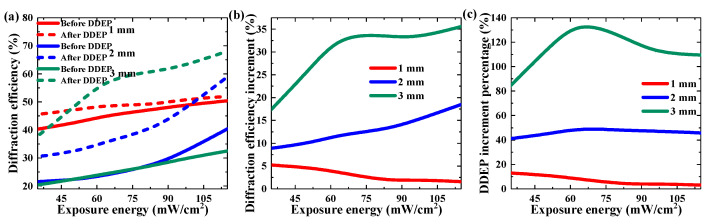
Effect of exposure energy on DDEP of materials with different thickness: (**a**) maximum diffraction efficiency, (**b**) diffraction efficiency increment, (**c**) DDEP increment percentage.

**Figure 5:**
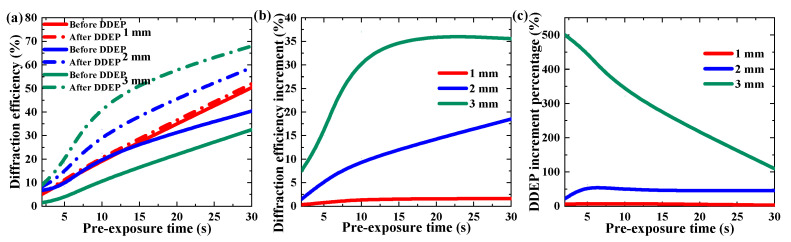
Effect of pre-exposure time on the DDEP of materials with different thickness: (**a**) maximum diffraction efficiency, (**b**) diffraction efficiency increment, (**c**) DDEP increment percentage.

The DDEP could improve the diffraction efficiency and holographic stability of TI/PMMA polymers without additional operations. In the experiment, we firstly examined the DDEP by recording a single grating under long-time (second level) exposure. Two factors, exposure energy and pre-exposure time, were investigated to analyze their influence on the DDEP, as shown in Figure 4 and Figure 5. In order to trigger the dark diffusion, we were required to pre-expose the sample in order to activate the photopolymerization reaction and form a concentration difference in the exposure area. Figure 4 depicts the influence of exposure energy on diffraction efficiency in the dark diffusion by maintaining the same pre-exposure time of 30 s. Here, the diffraction efficiency was defined as the ratio of diffracted intensity to transmission intensity in the reconstructing process. The exposure energy range was from 32 to 115 mW/cm^2^. With the increase in exposure energy, the diffraction efficiencies of the 2 mm and 3 mm samples improved by 2.12-fold (from 8.7% to 18.5%) and 2.25-fold (from 15.8% to 35.6%), respectively, while diffraction efficiency in the 1 mm sample declined by 70% (from 5.3% to 1.6%). Meanwhile, the diffraction efficiency increment was defined as the difference between the diffraction efficiency before and after dark diffusion enhancement, and the DDEP increment percentage was defined as the ratio of the diffraction efficiency increment in the dark diffusion to the original diffraction efficiency in post-exposure. As shown in Figure 4c, the DDEP increment percentages of the 1–3 mm TI/PMMA samples were 3.2–13.3%, 40.5–45.8% and 86.5–138.5%, respectively. This result illustrates that holographic grating strength in the thicker samples was mainly improved by the diffusion effect, while in thinner samples it was mainly enhanced by polymerization reactions.

Figure 5 depicts the influence of pre-exposure time on the diffraction efficiency in dark diffusion with the same exposure energy, 115 mW/cm^2^. With the pre-exposure time increasing, the diffraction efficiency enhancements of the 1–3 mm samples were 4-fold (from 0.4% to 1.6%), 12.3-fold (from 1.5% to 18.5%) and 4.8-fold (from 7.5% to 35.6%), respectively. However, we found that the DDEP increment percentage of the 3 mm sample decreased from 500% to 110% when the pre-exposure time increased from 2 s to 30 s. The results imply that with the increment in pre-exposure time, the contribution of photopolymerization to the grating formation gradually increased.

**Figure 6:**
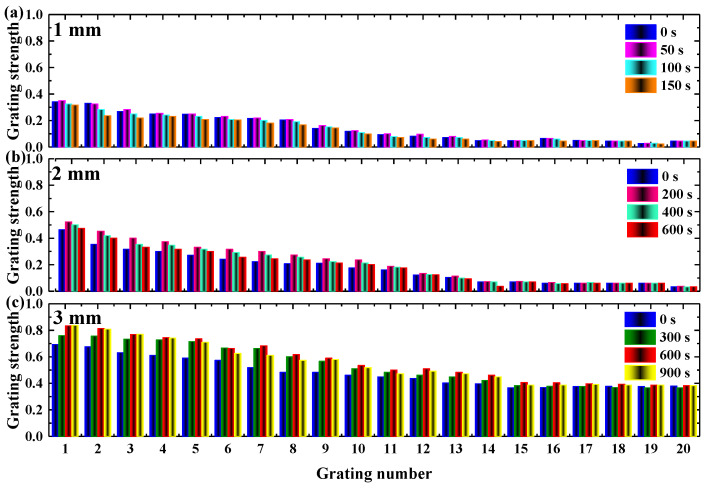
Enhancement of multiplexed gratings in dark diffusion: (**a**) 1 mm, (**b**) 2 mm, (**c**) 3 mm.

Then, we examined the storage capacity of the pre-exposure and the DDEP using angular-multiplexed gratings recording on the TI/PMMA polymers. We selected 5 s, 10 s and 20 s as the pre-exposure times for the 1–3 mm TI/PMMA polymers with the same exposure energy, 115 mW/cm^2^. In the experiment, we recorded 20 holographic gratings at the same position by controlling the electronical rotator with an accuracy of 1 degree, as shown in Figure 6. We took the grating strength of 0.2 as the benchmark. It was found that the number of multiplexing gratings above the benchmark was 8, 11, 20 in the 1–3 mm samples, respectively. This indicated that developing sample thickness could improve both storage density and recording quality. In addition, we investigated the stable multiplexing grating strengths during the DDEP [32], and the peak diffraction efficiency was achieved at 50 s, 200 s and 600 s in the 1–3 mm thick samples, respectively, which implied that the dark diffusion effect was closely related to the thickness of the recording medium, as shown in Figure 6.

**Figure 7:**
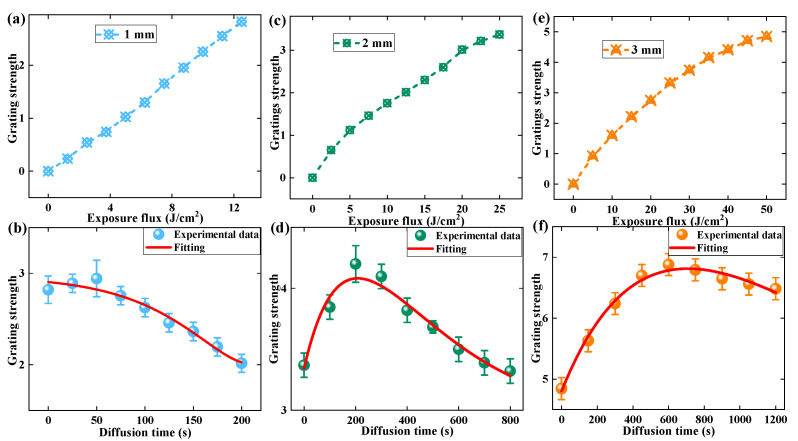
The evolution of grating strength during the pre-exposure and dark diffusion process: (**a**,**b**) 1 mm, (**c**,**d**) 2 mm, (**e**,**f**) 3 mm.

To investigate further, we measured the total multiplexing grating strengths before and after dark diffusion, as shown in Figure 7. The original grating strengths during the post-exposure process of the 1–3 mm samples were 2.82, 3.37 and 4.85, respectively. During the dark diffusion process, the peak grating strengths of the 1–3 mm thick materials were 2.94, 4.20 and 6.88. The corresponding increment ratios were 4%, 25% and 42%, respectively. Meanwhile, the stabilized grating strengths at the end of the dark diffusion were 2.02, 3.32 and 6.48. The corresponding attenuation ratios were 31%, 21% and 6%. This result indicates that thicker materials contributed high multiplexing capacity and stable storage ability. The lengths of error bars shown in Figure 7b,d,f were 0.1–0.2, 0.05–0.15 and 0.18, respectively.

### 3.3. Dark Diffusion Enhancement Process (DDEP) in TI/PMMA Polymers with Short-Time Exposure

**Figure 8:**
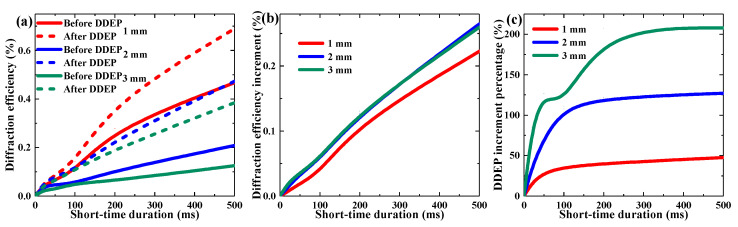
Effect of short-time duration on single shot DDEP of materials with different thickness: (**a**) maximum diffraction efficiency, (**b**) diffraction efficiency increment, (**c**) DDEP increment percentage.

After investigating the DDEP at the condition of long-time pre-exposure of the TI/PMMA polymers, we tried another exposure method, using millisecond order duration aiming to realize a transient grating recording. Figure 8 depicts the diffraction efficiency in the dark diffusion process after single shot exposure with different short-time durations (20~500 ms). The exposure energy was set to 115 mW/cm^2^ at this time and the exposure duration was controlled by an electronical shutter. It became evident that TI/PMMA is a type of sensitive photopolymer that is able to generate a dark diffusion effect even after pre-exposure of 20 ms. With the increment in the single shot duration, the contribution of the dark diffusion to the grating formation also rose. We achieved 48%, 127% and 207% DDEP improvement in the 1–3 mm samples using a pre-exposure of 500 ms.

**Figure 9:**
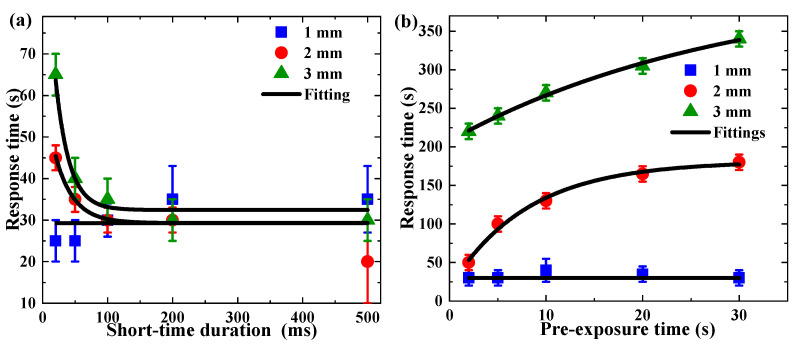
The response time in DDEP: (**a**) single shot with different short-time durations, (**b**) long-time pre-exposure with different times.

However, when we examined the response time of a single shot DDEP, a different trend was found compared with the impact of long-time exposure, as shown in Figure 9. Here, we defined the response time as the exponential fitting constant in the temporal evolution of the diffraction efficiency [33]. After single shot exposure, the response time declined with the increment in the single shot duration. Conversely, the response time to long-time post-exposure improved by raising pre-exposure time. The variation in the response time trend indicates that dark diffusion enhancement after short-time exposure made little contribution to grating strength, and the main reason for the generation of holographic grating was the photopolymerization reaction. In order to match the fitting curves, the length of error bars was 3–10 s in Figure 9a, and 10 s in Figure 9b.

**Figure 10:**
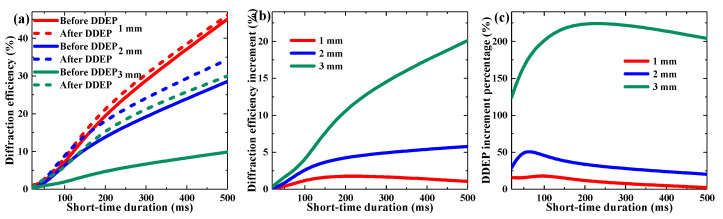
Effect of exposure time with different short-time durations on multi-shots DDEP of materials with different thickness: (**a**) maximum diffraction efficiency, (**b**) diffraction efficiency increment, (**c**) DDEP increment percentage.

**Figure 11:**
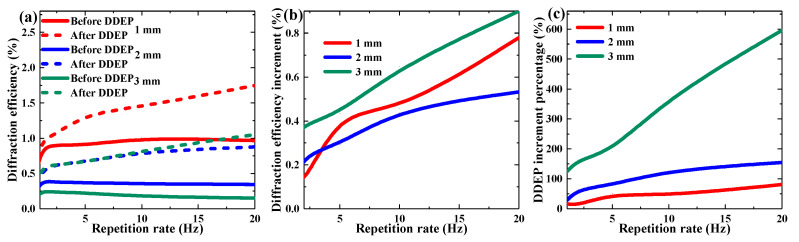
Effect of repetition rate on multi-shot DDEP of materials with different sample thickness: (**a**) maximum diffraction efficiency, (**b**) diffraction efficiency increment, (**c**) DDEP increment percentage.

To improve the grating strength in the dark diffusion process, we adopted multiple short-time exposures, as shown in Figure 10 and Figure 11. Each recording position was exposed 20 times, with different single shot durations and repetition rates, respectively. Figure 10 describes the influence of single shot duration on grating strength enhancement using multiple short-time exposures. It was found that the grating enhancement of the DDEP could be developed by increasing exposure times and single shot duration, which implied that the holographic properties of TI/PMMA could be adjusted by controlling pulse exposure rates and durations.

Figure 11 depicts the DDEP in multiple short-time exposures using different repetition rates. When the total exposure flux was equal (exposure 20 times to an exposure energy of 115 mW/cm^2^), we varied the exposure repetition rate within the range of 1~20 Hz. It was found that the repetition rate influenced both the pre-exposure process and the dark diffusion process. Compared with the dark diffusion, the influence of the pre-exposure gradation upon grating formation was slight. For example, in the 3 mm TI/PMMA sample, the grating strength generated during the pre-exposure process with different repetition rates was between approximately 0.15 and 0.26%, while the grating enhancement in dark diffusion improved from 124% to 596%. By increasing the exposure repetition rate, the dark diffusion effect can be enhanced, while it is possible to weaken the photo-polymerization effect. Exposure repetition rate seems to be an effective method to control the proportion of dark diffusion in the grating formation process.

## 4. Conclusions

The dark diffusion effect is an important characteristic of photopolymers used in holographic storage under dark conditions without any additional operations. Overall, we investigated the dark diffusion properties of TI/PMMA polymers experimentally and theoretically. The post-exposure process was mainly determined by two aspects, the diffusion of photo-initiators and the anti-diffusion of photoproducts, which led to the enhancement and attenuation of holographic grating strength. In the experiment, we adopted two approaches, consecutive (long-time) and short-time exposures, to examine the effect of dark diffusion in our materials, TI/PMMA polymers. In the first part, single and multiplexing gratings were recorded in materials under long-time exposure (second level). We investigated the influence of exposure energy (32–115 mW/cm^2^) and exposure time (2–30 s) on the dark diffusion effect in TI/PMMA polymers. The DDEP could be enhanced by increasing the exposure duration and energy. We achieved a 35.6% diffraction efficiency enhancement and 4.8-fold grating contribution ratio in the 3 mm sample. Furthermore, we obtained a cumulative grating strength of 6.88 and corresponding increment ratio of 42% using angular multiplexed storage. In the second part, a short exposure (millisecond level) was applied to examine the DDEP of TI/PMMA polymers. We achieved a 200% diffraction efficiency improvement through dark diffusion in the 3 mm sample after 500 ms pre-exposure. Meanwhile, the response time during the DDEP declined with the increase in the single-shot exposure duration. The influence on the DDEP of single shot duration and repetition rate under multishot exposure was researched in detail. The grating enhancement induced by dark diffusion could be improved by raising the single-shot duration and repetition rate. This research can promote the realization of holographic storage applications using our bulk TI/PMMA polymers via the dark diffusion method.

## Data Availability

The data presented in this study are available on request from the corresponding author.
